# Gene silencing in adult *Popillia japonica* through feeding of double‐stranded RNA (dsRNA) complexed with branched amphiphilic peptide capsules (BAPCs)

**DOI:** 10.3389/finsc.2023.1151789

**Published:** 2023-05-12

**Authors:** Elijah Carroll, Nitish Kunte, Erin McGraw, Sujan Gautam, Ryan Range, Jose A. Noveron-Nunez, David W. Held, L. Adriana Avila

**Affiliations:** ^1^ Department of Entomology and Plant Pathology, Auburn University, Auburn, AL, United States; ^2^ Department of Biological Sciences, Auburn University, Auburn, AL, United States

**Keywords:** Japanese beetle, *Scarabaeidae*, dsRNA (double-stranded RNA), pest management, nanoparticles, oral delivery

## Abstract

Gene silencing by feeding double-stranded (dsRNA) holds promise as a novel pest management strategy. Nonetheless, degradation of dsRNA in the environment and within the insect gut, as well as inefficient systemic delivery are major limitations to applying this strategy. Branched amphiphilic peptide capsules (BAPCs) complexed with dsRNA have been used to successfully target genes outside and inside the gut epithelium upon ingestion. This suggests that BAPCs can protect dsRNA from degradation in the gut environment and successfully shuttle it across gut epithelium. In this study, our objectives were to 1) Determine whether feeding on BAPC-dsRNA complexes targeting a putative peritrophin gene of *P. japonica* would result in the suppression of gut peritrophin synthesis, and 2) gain insight into the cellular uptake mechanisms and transport of BAPC-dsRNA complexes across the larval midgut of *P. japonica.* Our results suggest that BAPC-dsRNA complexes are readily taken up by the midgut epithelium, and treatment of the tissue with endocytosis inhibitors effectively suppresses intracellular transport. Further, assessment of gene expression in BAPC- peritrophin dsRNA fed beetles demonstrated significant downregulation in mRNA levels relative to control and/or dsRNA alone. Our results demonstrated that BAPCs increase the efficacy of gene knockdown relative to dsRNA alone in *P. japonica* adults. To our knowledge, this is the first report on nanoparticle-mediated dsRNA delivery through feeding in *P. japonica*.

## 1 Introduction

Invasive species account for significant ecological and economic impacts (1, 2). In 1916, a small, metallic-colored beetle from Japan, *Popillia japonica* Newman, was first detected near Riverton, NJ, USA*. P. japonica* is currently established in 28 states in the US, and continues to expand its range west and north in North America into previously non-infested states, territories, and provinces, likely through human-mediated transport (3, 4). The polyphagous nature, feeding on >300 plant species, and capable of forming large feeding aggregations on host plants (5, 6) are key factors in the success of *P. japonica* adults as pests in the extended geographic range. This species is a target for substantial insecticide usage in both larval and adult life stages, especially in areas with large monocultures of turfgrass such as roadsides, golf courses, and urban landscapes (6). Insecticide use targeting *P. japonica* adults and larvae are associated with secondary pest outbreaks (7) and interference with host finding by introduced natural enemies (8).

Targeting insect pests, especially beetles, with double-stranded ribonucleic acid (dsRNA) molecules has shown great promise as an alternative to chemical insecticides (9–11). Exogenous dsRNA activates the RNA interference (RNAi) pathway, which is a conserved and innate biological defense mechanism in eukaryotic organisms against viruses and transposons *via* post-transcriptional gene silencing (12). Unlike common chemical insecticides, dsRNAs are designed to target mRNA sequences unique to the target pest because of the necessity to have high sequence fidelity for gene silencing to occur (9). Furthermore, dsRNA has a low potential for persistence in the environment, including soil, sediment, and surface water compartments, because of its instability in environmental conditions and rapid microbial degradation (9, 13, 14).

The most field-applicable route of dsRNA delivery is *via* ingestion by the target insect (15). So far, the only commercially available dsRNA product for insect control is facilitated by genetically modified plants (10). However, these products involve plant transformation, which is not feasible for all plants/crops due to the cost and time of production, and extensive regulatory processes to evaluate environmental risk (16). Thus, exogenously applied products in the form of bio-pesticides may be a more feasible and cost-effective method for pest attacking multiple plant/cropping systems. The general use of dsRNA as an insecticide has been forestalled by the variability in efficacy of RNAi among species, life stage, dosage, delivery method, and target gene (15, 17–19). The observed variability in efficacy may be contributed to degradation of dsRNA in the environment and in the insect gut, inefficient uptake by the gut epithelium, defective RISC complex, and impaired systemic delivery (15, 20–22).The enzymes present in body fluids of *P. japonica* are highly efficient at degradation of dsRNA relative to other beetles that have been successfully targeted (23). Hence, it is imperative to provide a protectant to dsRNA for silencing effects to occur.

Nanoparticles can help to overcome the technical challenges associated with the oral delivery and efficiency of dsRNA. Nanoparticles are typically defined as particles ranging between 1 and 100 nm in size made of a variety of materials (i.e., lipids, peptides, polymers and metals) (15, 24). In most cases, nanoparticle/dsRNA complexes are formed by electrostatic interactions between the cationic groups of the nanoparticles and the negatively charged phosphate groups of dsRNA (25). Nanoparticles can prevent degradation of the dsRNA nucleotides by nucleases in the salivary glands and in the gut by blocking target sites for RNases (26, 27). Furthermore, the overall net charge of the complexes are typically positive, which is suitable for uptake by cell membranes (25, 28). Our research team developed branched amphiphilic peptide capsules (BAPCs) (25). BAPCs are formed through the spontaneous assembly of two branched amphiphilic peptides, bis(Ac-FLIVI)–K–K_4_–CONH_2_ and bis(Ac-FLIVIGSII)–K–K_4_–CONH_2_in water (29). Ingestion of BAPC-dsRNA complexes targeting a major genes associated with the unfolded protein response resulted in significant knockdown of gene expression levels and mortality rates in the red flour beetle (*Tribolium castaneum*) and in the pea aphid (*Acyrthosiphon pisum*) (25). The properties of BAPCs also make the synthesis scalable to large scale production as they can be stored for extended periods, self-assemble in pure water, and are effective at low μM concentrations.

In this study, BAPC-dsRNA complexes were evaluated for their efficiency in 1) knockdown of the peritrophin expression and subsequent mortality post-ingestion in adult *P. japonica*, and 2) uptake and transport across the larval midgut epithelial cells. Silencing of peritrophin can make insect gut more susceptible to insecticides, phytochemicals and pathogens affecting their metabolism, growth, development, and survival (30). We also analyzed BAPCs and BAPC-dsRNA size in a buffered solution with pH ~7.4 (similar to *P. japonica* midgut). Finally, we explored the cellular mechanism for uptake of BAPC-dsRNA complexes and transport across larval midgut (30). According with the literature review, this is the first report on gene knockdown in adult *P. japonica* using nanoparticle-mediated dsRNA delivery.

## 2 Methods

### 2.1 Specimens

Larvae of *P. japonica* were sourced from a commercial sod farm near Murfreesboro, TN and collected in April 2022. Larvae used for midgut assays were maintained by placing them in individual cells of ice cube trays and fed with carrot strips. In July 2022, field collected, adult female *P. japonica* used in the dsRNA feeding trials were shipped overnight from Michigan State University. The beetles were maintained in a container with sifted soil and fed on a diet of crape myrtle (*Lagerstroemia indica*) and rose (*Rosa* spp.) flowers. Female and male beetles were separated based on the morphology of the tibial spur and only insects that were free of obvious morphological defects or injuries were used in experiments (5).

### 2.2 Preparation of BAPCs and BAPC-dsRNA complexes

To form the BAPCs, two monomeric peptides, bis(Ac-FLIVI)-K-K_4_-CONH_2_ and bis(Ac-FLIVIGSII)-K-K_4_-CONH_2_, were synthesized using solid phase peptide synthesis as previously described by Avila et al. (2018) (25). These peptides are referred to as H5 and H9, respectively, after the number of residues in the hydrophobic branches. After synthesis, dried peptide was dissolved in trifluoroethanol (TFE). Concentration of each peptide was determined by measuring the absorbance of phenylalanine and subsequently dividing that value by two to account for the two phenylalanine residues per peptide. The two peptides were then mixed in equimolar ratios to create a 1mM final stock. TFE was then evaporated off using a FreeZone2.5 and refrigerated Centrivap Concentrator vacuum system (LabConco). BAPCs were assembled by adding 1 mL nuclease-free water and allowing the solution to sit at room temperature for 5 min, followed by an incubation at 4°C for at least 1 hr. At room temperature, the peptides spontaneously assemble into a bilayer and fuse, and the shift to 4°C slows the fusing of complexes and locks the BAPCs in a size range of 50-250 nm. At room temperature, the peptides spontaneously assemble into a bilayer and fuse, and the shift to 4°C slows the fusing of complexes and locks the BAPCs in a size range of 50-250 nm. BAPC-dsRNA complexes were formed by mixing the appropriate concentration of BAPCs with 1μg dsRNA and allowing the mixture to sit for 15 min at room temperature.

To form rhodamine-labelled BAPCs (Rh-BAPCs), half of the bis (Ac-FLIVI)-K-K_4_-CONH_2_ component was substituted with the same peptide labeled with the N-hydroxysuccinimide ester of rhodamine B covalently attached to the ϵ-amino group of the C-terminus lysine (bis (Ac-FLIVI)-K-K_3_-K(Rh)-CONH_2_). This resulted in a final molar ratio of 1 H9: 0.5 H5: 0.5 Rh-H5. Rh-BAPC-dsRNA complex were formed as described for unlabeled BAPC-dsRNA complexes.

### 2.3 Dynamic light scattering and electrophoretic retardation assay of BAPCs and BAPC-dsRNA complexes 

BAPCs and BAPC–dsRNA complexes were suspended in a buffer simulating midgut pH, then size was measured *via* DLS using the Zetasizer Nano ZS (Malvern Instruments Ltd., Westborough, MA). A 500μM stock of BAPCs was prepared following the protocol previously described. BAPCs were then complexed with 1 µg of dsRNA if needed, and the BAPCs or BAPC-dsRNA complexes were transferred to phosphate buffered saline solution (pH = 7.4). Samples were incubated at room temperature for 5-10 minutes prior to analysis, and all DLS measurements were performed in triplicates. For the gel retardation assay, BAPC-dsRNA complexes of 20μM and 60μM BAPCs complexed with 1μg dsRNA were assembled and incubated in pH 7.4 buffer as described above. Following, complexes were mixed with RNA gel loading dye (Invitrogen, Waltham, MA) at a 1:1 ratio. Samples were then resolved onto a 2% agarose gel composed of 1× MOPS buffer and SYBR green stain, then electrophoresed at 100 V for 30 min. Control wells containing 1 μg of dsRNA only and BAPC concentrations of 20µM and 60 µM without dsRNA were included. The gel was imaged using ImageQuant LAS 4000 (GE Healthcare, Pittsburgh, PA, USA).

### 2.4 Selection of target gene and dsRNA synthesis

Due to availability of only five known mRNA sequences of *P. japonica* in NCBI database, we chose peritrophin, one of the available sequences, as a target for RNAi. Peritrophins play key protective roles during food processing in feeding life stages, growth, and development of larvae. To synthesize dsRNA, first total RNA was extracted from the gut tissue of *P. japonica* using a commercially available TRIzol reagent. After purification of total RNA, the RNA was reverse transcribed to cDNA using SuperScript II First-Strand Synthesis System, and the genomic DNA was removed by DNase I treatment (Invitrogen). The synthesized cDNA was then used as a template for the amplification of the peritrophin gene segment using following primers; forward primer: GCTGGTACCTACTTCAATCC, reverse primer: CATACAACCTGCATCTTCGG. Both primers were designed manually to amplify the peritrophin gene segment of ~300 bps with T7 promoter sequence flanking at 5’ end of both primer sequences. Upon amplification and purification of T7 flanked peritrophin DNA, sense and antisense RNA strands were synthesized separately as per manufacturer protocol using TranscriptAid T7 High Yield Transcription Kit (Thermo Scientific™, Carlsbad, CA, USA). After transcription, the sense and antisense single-stranded RNA (ssRNA) were purified using LiCl precipitation, quantified using nanodrop and resuspended in duplex buffer (Integrated DNA Technologies Inc., USA). For annealing, both RNA strands were mixed in 1:1 molar ratio and annealed as recommended by the supplier. The quality of dsRNA was verified by 1% agarose gel electrophoresis and using a Nanodrop technique. In addition, we synthesized dsRNA sequence non-specific to *P. japonica* and used as a non-specific control.

### 2.5 Adult feeding and survivorship assay

One day prior to the experiment, 120 adult female beetles were randomly selected from a container and transferred to a plastic cup (Dart, Mason, MI) and deprived of food for 24 hr prior to feeding. Whatman GF/A filter papers (25 mm diam., Cytivia life sciences, Marlborough, MA) were cut into quarters and pinned between two 5 x 5 mm pieces of transparency film (Tri-state Visual Products, Highland Heights, KY) using stainless steel insect pins. Each filter paper quarter was treated with 40 µL of a 1 M sucrose solution and allowed to dry for 12 hr prior to applying the treatment to promote adult beetles feeding. Adult *P. japonica* will feed on filter papers amended with 1 M sucrose (31). Upon drying, filter papers for seven different treatment groups and 15 biological replicates were prepared by applying 40 µL of BAPC-dsRNA complexes.

On day 0, 100 food-deprived beetles were selected for the survivorship assay. Individuals were tested for vigor by flipping them on their dorsum and used only if they could right themselves within 5 min. Selected beetles were then transferred into an individual wax-bottomed plastic cup and randomly assigned to one of the seven treatments. Three extra beetles were randomly selected, deprived of food for 24 hr and their midgut tissues were isolated and preserved in TRIzol to assess effect of starvation on peritrophin gene expression. Once the beetles were transferred to wax-lined cups, filter paper quarters with BAPC complexes were placed inside and all beetles were transferred into a growth chamber at 25 ± 0.5° C. Beetles were allowed to feed until a treatment group had consumed either an average of 2/3 (66%) of the filter paper or 24 hr whichever occurred first. Post feeding, all filter papers were replaced with leaf disks (20 mm diam.) taken from freshly collected Virginia creeper (*Parthenocissus quinquefolia*) foliage, followed by replacement of these leaf disks daily until day 6. Until the end of experimental protocol or observed mortality, beetles were tested daily for vigor as previously described. Beetles that failed to vigor response were considered dead and eliminated from the study. Three beetles from each group were selected at random and dissected to isolate gut tissue for RNA extraction and analysis.

On day 7, a second dose of respective treatments was administered to surviving beetles through filter papers using the same methodology as described above. Data on survivorship was collected every 24 hr until day 14. Filter paper consumption was calculated using Image-J (32) by collecting the filter papers quarters after both doses. The area of filter paper after the assays was measured then a percentage of area consumed was calculated based on the initial area. The initial area was the average of six filter paper quarters not provided to beetles.

### 2.6 Quantitative polymerase chain reaction

Total RNA was isolated from adult *P. japonica* gut tissue with TRIzol Reagent (TRIzol (Ambion, Inc., Carlsbad, CA, USA). RNA concentration was measured using nanodrop and quality was evaluated using 260/280 and 260/230 ratio. cDNA was synthesized using SuperScript II Reverse Transcriptase (Invitrogen, Carlsbad, CA) according to manufacturer instructions and used as a template for the RT-qPCR. Each RT-qPCR sample contained 10 µL of synthesized cDNA, 0.8 µL of each primer (10 μM forward and reverse), 0.9 µL of nuclease free ddH2O, and 12.5 µL of Perfecta Sybr Green Supermix (Quanta Biosciences, Gaithersburg, MD, USA); totaling 25 µL. All reactions were performed using SYBR Green Master Mix and amplified under the following cycling conditions: beginning cycle at 95°C, 40 cycles at 95°C for denaturation, followed with 30 s at 65°C for annealing and extension, and ending with generation of a melting curve consisting of a single peak to rule out non-specific product and primer dimer formations. Each treatment group contained three biological replicates and two technical replicates. The expression levels of peritrophin and the number of transcripts per sample was estimated based on the Ct value. Due to unavailability of housekeeping gene sequences in *P. japonica*, we used β-actin gene sequence from the closely related species *Oryctes rhinoceros* (Coleoptera: Scarabaeidae) to design primers and used as an internal loading control. Generated cDNA was then used to quantify changes in gene expression levels among different treatment groups by RT-qPCR. The expression levels of the genes were determined by 2−ΔΔCt* proportional calculation method. (The fold changes in peritrophin transcript levels relative to the β-actin). For statistical analysis, we performed a one-way ANOVA with Tukey’s post-test to evaluate differences between treatment group *P < 0.05.*

### 2.7 Larval midgut transcytosis assay

To elucidate if transcytosis was involved in the translocation of BAPCs through midgut epithelium cells, live third instar *P. japonica* larvae were dissected to isolate the midgut tissues. The preparation for assays in the Ussing chamber have been described in our previous work (29). Larvae used in this assay were stored in a fridge at 4°C for 48 hr prior to dissections to stabilize the gut tissues. Incisions were made along the lateral-medial line of the larvae from the anterior to the posterior using corneal scissors. Insect pins were then used to secure the integument onto the dissection tray and expose the digestive tract. Incisions were performed on the midgut by making lateral-medial incisions anteriorly from the third gastric caecum. The tissues were then isolated from the body, rinsed with insect physiological solution described previously (29), and immediately mounted onto a modified 0.01 cm^2^ slide. Dissected midgut tissue from larvae was inserted into a tissue holder slide which was placed inside of a Ussing chamber. This chamber creates an *ex vivo* gut environment through which transport of molecules across tissue may be studied. Buffer containing rhodamine dye labelled BAPCs was added to the luminal side of the tissue, and transcellular transport was determined by measuring rhodamine dye fluorescence on luminal as well as hemolymph side at discrete time points. Dissections were conducted with meticulous effort to avoid tissue punctures and to conserve orientation of the tissue relative to the lumen and the hemolymph.

After mounting, slides were slotted into the two-sided chamber, where tissues were then perfused with 3 mL of lumen or hemolymph buffer according to tissue orientation (29). To study the effect of inhibiting endocytosis on the transport of BAPC-dsRNA complexes, three replicate tissues were pre-incubated with 10µM chlorpromazine (CPZ) for 30 min before the addition of Rh-BAPC-dsRNA complexes. A final concentration of 50 µM Rh–BAPCs with or without 1 µg dsRNA was then added to the lumen side. Tissues were exposed for a period of 120 min, after which fluorescence was read using a BioTek Cytation 3 plate reader (λex = 544 nm; λem = 576 nm). Change in relative fluorescence over time was plotted to visualize the transport of Rh-BAPCs due to transcytosis. Fluorescence was measured in arbitrary fluorescence units (AFU’s). To account for the variability of relative fluorescence between replicates, data were normalized using proportions.

### 2.8 Statistical analysis

Data analysis and plots were done using GraphPad Prism (version 8.0.0 for Windows, GraphPad Software, San Diego, California USA). To estimate the sample size in the survivorship assay, we employed the “resource equation method” (33). For survival curves we used the Log Rank Test. For the gene expression analysis, consumption of filter papers, and DLS experiments, we used one-way ANOVA using Tukey as post-test. Transcytosis experiments were analyzed using two-way ANOVA and Tukey as post-test. An alpha level of *P<0.05* was used for all analyses.

## 3 Results

### 3.1 *P. japonica* artificial diets supplemented with BAPC-dsRNA complexes

Males often perform less consistently in feeding assays relative to females because their behavior is more directed toward mating (31). Thus, we included adult females exclusively to evaluate the efficiency of the BAPCs to deliver dsRNA targeting peritrophin through feeding. As described in the methods section, the BAPC-dsRNA complexes were applied on filter-paper containing sucrose, a common phagostimulant used in *P. Japonica* assays (34, 35). The potential of BAPCs to deliver dsRNA in *P. japonica* was evaluated through survival assay and gene expression analysis.

**Figure 1:**
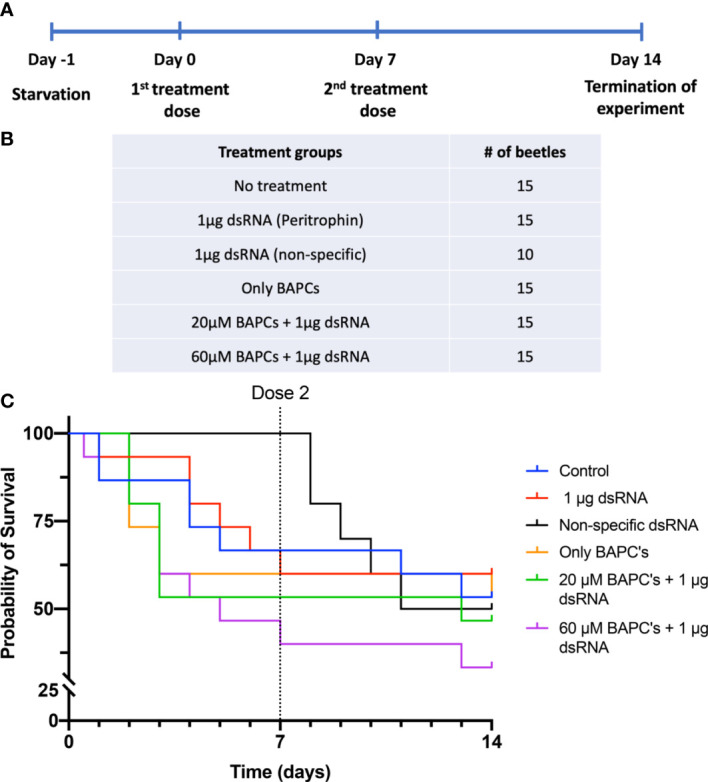
Survival curves of *P. Japonica* post-ingestion of BAPCs formulations: Experimental outline of dsRNA feeding assay in *P. japonica* adult females **(A)**. Treatment groups included in the feeding assay **(B)**. The survival curve of *P. japonica* females upon feeding on BAPCs complexed with peritrophin-dsRNA (n = 15) **(C)**. The data were analyzed using a log rank test. There were no significant differences between treatment groups (*P >0.05*).

We delivered two doses of BAPC-dsRNA complexes through feeding on day 0 and day 7, allowing them to feed on the treatment up to 24 hr (**Figures 1A, B**). Subsequently, we monitored all beetles daily for mortality up to 14 days (normal life span of adult *P. japonica* is 30-45 days). Survivorship by 14 days was only 33% in insects fed on diets containing 1 μg dsRNA+60 μM BAPCs (t_1/2 =_ 7 _d_). On the other hand, 60% and 53% survivorship were observed at 14 days in beetles fed only dsRNA and untreated control groups, respectively. This difference in survivorship was not significant as determined by a log-rank test (Day 7: *P= 0.12* & Day 14: *P = 0.6, df= 1*). Feeding of beetles on a diet of non-specific (non-peritrophin) dsRNA or with lower BAPCs concentration (1 μg dsRNA+ 20 μM BAPCs) also had no effect on survival (t_1/2 =_ 14 _d_) (**Figure 1C**).

Consumption of filter papers vary greatly among individuals within same treatment group, therefore the average dosage delivered would be lower than provided. We calculated surface area of filter paper consumed as a proxy for dose ingested within 24 hr before being replaced with a diet of Virginia creeper leaf discs. On average, 38% of the filters were consumed across treatments, with an average of 22% of the filter paper consumed in the control dsRNA treatment (**Supplementary Figure 1**) *(P<0.01, F = 3.087, Dfn =6, Dfd=139)*. Thus, less than 1 μg of dsRNA dose is sufficient to induce significant knockdown effects when delivered with the aid of an effective concentration of BAPCs.

### 3.2 Assessment of peritrophin-mRNA levels isolated from *P. japonica* midgut

**Figure 2:**
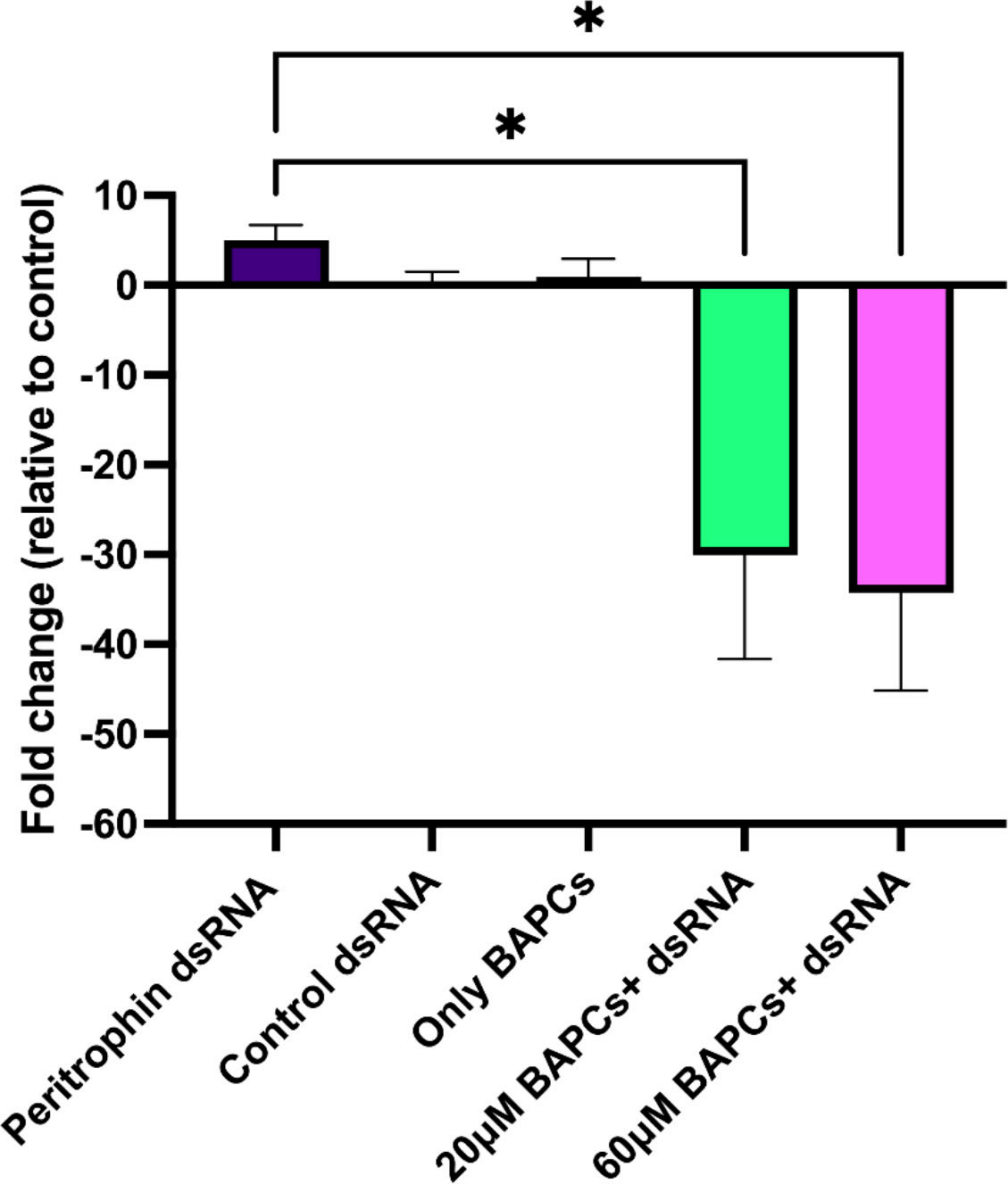
Analysis of gene expression upon dsRNA mediated gene silencing: peritrophin-mRNA transcript levels in the midgut of *P. japonica* upon feeding on BAPCs complexed with and without dsRNA analyzed using RT-qPCR. Fold change in peritrophin expression is normalized using β-actin as an internal control. Differences between values were compared by one way ANOVA using Tukey as post-test. Statistical significance: (*) *P < 0.05*; (**) *P < 0.01*. Non-statistical significance (*ns*) was considered when *P > 0.05*.

We quantified the peritrophin transcript levels by RT-qPCR analysis to confirm that dsRNA induced gene silencing in the targeted gene. Ingestion of 20 μM BAPCs+ 1 μg dsRNA resulted in a 30-fold decrease in peritrophin gene expression, which was significantly different *(P<0.05, F= 6.840, Dfn= 4, Dfd= 10)* from the dsRNA alone group. Similarly, 60 μM BAPCs+ 1 μg dsRNA had the greatest gene silencing rate, with knockdown of expression by approximately 34-fold relative to non-treated control group (**Figure 2**). Although, quantification of the mRNA transcripts is congruent with the trends observed in the survivorship study, our results support the concept of BAPCs nanoparticles acting as dsRNA stabilizer, and cellular uptake enhancer. Furthermore, we also analyzed the integrity of the BAPCs formulations by measuring the size in a buffered solution with a pH similar to the *P. japonica* midgut (pH=7.4) (5).

### 3.3 Biophysical characterization of BAPCs and BAPC-dsRNA complexes

**Figure 3:**
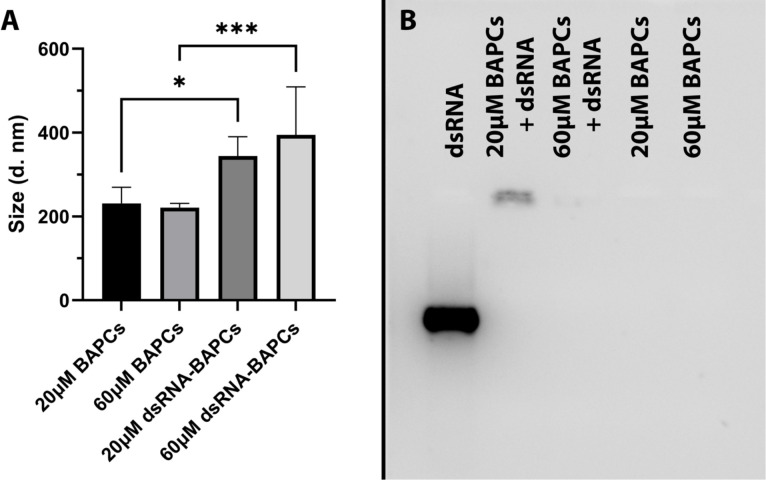
**(A)** Stability assessment of BAPCs and BAPC-dsRNA complexes in a buffer mimicking *P. japonica* gut (pH 7.4). **(B)** BAPC loading capacity assessed by the electrophoretic mobility shift assay. BAPC-dsRNA complexes were formed by mixing 20μM and 60μM BAPCs with 1μg dsRNA. Controls containing only 20μM or 60μM BAPCs without dsRNA were also run to show they did not produce background signal. Differences between values were compared by one way ANOVA using Tukey as post-test. Statistical significance: (*) *P < 0.05*; (***) *P < 0.001*. Non-statistical significance (ns) was considered when *P > 0.05*.

From a biophysical perspective, the stability or integrity of nanoparticles is used to describe the preservation of a particular nanostructure property (i.e., size). We assessed BAPCs stability by incubating in buffer of pH 7.4 using dynamic light scattering (DLS), According with **Figure 3A**, the BAPCs–dsRNA complexes displayed a size ranging between 250 to 350 nm, a size that is consistent with our previously reported DLS measurements performed in distilled water (29). BAPCs (60 μM) not associated with dsRNA exhibited a significant *(F = 10.45, P= 0.0002, Dfn=3, Dfd=20)* smaller hydrodynamic diameters than the BAPC-dsRNA complexes, confirming that the association of dsRNA increases the size of the BAPCs or causes BAPCs to cluster together. Furthermore, these results indicate that the complexes do not dissociate or aggregate in the buffered solution, proving structure stability in a pH environment consistent with the gut of *P. japonica *adults. Although multiple variables can play a role in nanoparticle stability inside the midgut, the pH is critical since it can lead to variation in nanoparticle charge and oxidation state. Regarding nuclease degradation of dsRNA, studies performed in mammalian cells support the notion that BAPCs protect dsRNA against nuclease degradation (29, 36). Target sites of RNAs might no longer be accessible to the catalytic core of RNases after the association with the BAPCs surface (37). It is important to mention that nucleases exclusively affect the dsRNA structure and not BAPCs. The downregulation of the peritrophin transcript levels also support the notion of nuclease protection conferred by BAPCs.

To elucidate the dsRNA binding capacity of BAPCs at concentrations used for the survivorship assay, we evaluated their electrophoretic mobility in a 2% RNA agarose gel. Our results indicated that association of dsRNA with the BAPCs surface led to a decreased migration of dsRNA that was dependent on BAPC concentration (**Figure 3B**). The formulation with the highest BAPCs concentration (60 μM) displayed a barely visible band, suggesting that all added dsRNA has firmly adhered to the BAPCs surface, which resulted in a poor interaction with the dye SYBR green. However, lower concentrations of BAPCs yielded a more visible dsRNA band, indicating more availability for SYBR green binding due to a weaker interaction of BAPCs and dsRNA. As expected, BAPCs not complexed with dsRNA showed no signal in the well.

### 3.4 Midgut cellular uptake mechanisms of BAPC−dsRNA complexes

**Figure 4:**
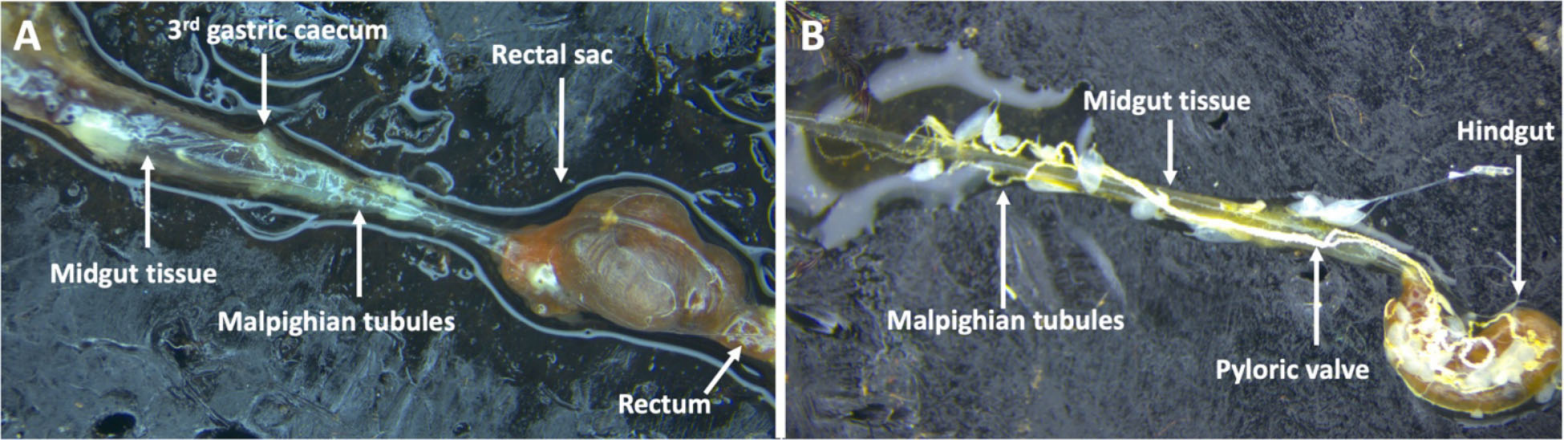
Digestive tract of *P. japonica*. **(A)** Larval digestive tract including midgut tissue, 3^rd^ gastric caecum, Malpighian tubules, rectal sac, and the rectum. **(B)** Adult digestive tract including midgut tissue, hindgut, the pyloric valve, and Malpighian tubules (White, string-like organ).

The alimentary tract of adult and 3^rd^ instar larval stages of *P. japonica* have only been described in separate publications with supporting hand illustrations (38, 39) respectively. More recent photo images of the digestive tract of neonate and 3^rd^ instar grubs have been published (40). Here, for the first time we present comparative images of the 3^rd^ instar larval and adult alimentary tracts juxtaposed to highlight the morphological differences (Gryphax^®^ Series Avior microscope camera, Jenoptic, Jena, Germany). These images confirm previous descriptions (38, 41) stating the adult midgut is narrower in width relative to the larval midgut. Through the many dissections for the reported experiments, we noted less tissue strength of the adult gut leading to those tissues tearing and shearing more easily than the larval midgut tissues (**Figures 4A, B**). The width and sensitivity of the adult midgut tissue was an obstacle to study BAPC-dsRNA complexes cellular uptake and transport across gut tissue. Consequently, only the larval midgut tissues were used to study these mechanisms in *P. japonica*. For larval dissections, we used the third gastric caecum to delineate between midgut and hindgut tissues (**Figure 4A**).

**Figure 5:**
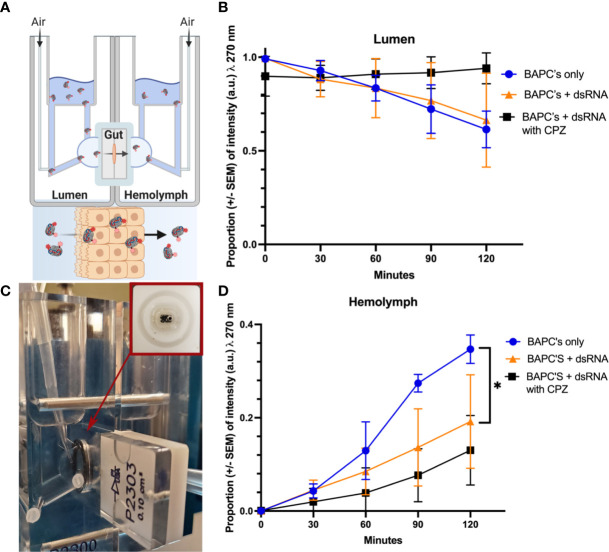
Cellular uptake study. Mechanism of Rh–BAPCs and Rh–BAPC-dsRNA cellular uptake by *P. japonica* midgut cells. **(A, B)** graphical representation and actual set up of Ussing chamber used for *ex vivo* analysis of BAPC-dsRNA complexes uptake and transport across *P. japonica* midgut tissue. **(C)** Mean relative fluorescence of Rh-BAPCs complexes on luminal side buffer and **(D)** Mean relative fluorescence of Rh-BAPCs complexes on hemolymph side buffer over 2 hr. Differences between values were compared by two-way ANOVA using Tukey as post-test. Statistical significance: (*) *P< 0.05*.

BAPCs and BAPC-dsRNA complexes transport across the gut tissue was assessed with the help of Ussing chamber (**Figures 5A, B**). Both formulations are actively transported across the gut tissue, with around 50% reduction in rhodamine fluorescence on luminal compartment (**Figure 5C**). However, BAPCs complexed with dsRNA slows significantly*(P<0.05, F= 5.841, Dfn=2, Dfd=6)*. the rate of transcellular transport compared to only BAPCs (**Figure 5D**). A plausible reason for the diminished transport can be related to the binding of the negatively charged dsRNA to the surface of BAPCs. The dsRNA association blocks a portion of the positively charged lysine residues exposed on the BAPCs surface thus becoming less cationic, and reducing cellular uptake by epithelial cells (42) (**Figure 5**).

To assess the role of endocytosis on the uptake of BAPC-dsRNA complexes, gut tissue was pre-incubated for 30 min with chlorpromazine, an inhibitor of clathrin-mediated endocytosis. Clathrin-mediated endocytosis is one of the major pathways by which cells transport extracellular cargo from outside the cell membrane to the interior *via* the formation of clathrin-coated endocytic vesicles. Following CPZ addition, Rh-BAPC-dsRNA complexes were added to luminal side, and the relative change in rhodamine fluorescence was measured as described earlier. As Rh-BAPCs are taken up from the luminal side, it is expected that fluorescence of this compartment will decrease. Subsequently, movement *via* transcytosis will deposit endocytosed Rh-BAPCs to the hemolymph compartment, increasing its fluorescence over time (29). Treatment of gut tissue with endocytic inhibitor abrogated BAPC-dsRNA uptake from luminal side with no change in rhodamine fluorescence up to 2h (**Figure 5C**). Similarly, there was no significant increase in rhodamine fluorescence was observed on hemolymph side (**Figure 5D**). It is also expected that a fraction of BAPCs or BAPCs-dsRNA remain trapped within the *P. japonica* gut cells, particularly in endosomal vesicles such as endosomes and lysosomes (43–45). Retrograde transport is also possible and has been documented for other nanoparticles (45). In other words, only some of the material that enters cells will undergo transcytosis.

Overall, these results indicate that cellular uptake of BAPCs and BAPC-dsRNA complexes is mediated by clathrin coated endocytic vesicle. Nonetheless, cellular uptake is a complex process and potentially other mechanisms can also be involved in the uptake of BAPCs formulations.

## 4 Discussion

In summary, we reported the first gene knockdown study in adult *P. japonica* by feeding of BAPC-dsRNA complexes. Although there was a numerical (20%) difference in survivorship between the 60 μM BAPCs +1 μg of peritrophin-dsRNA treatment and the non-treated control group, no statistical differences were observed between treatment groups. Here, we discuss the potential reasons for the lack of observed mortality in groups that ingested dsRNA. Peritrophic matrix proteins (PMP’s) and analogs are diverse in Coleoptera, and few have been experimentally demonstrated to have significant effects on the structure and function of the peritrophic matrix. A total of 11 genes encoding PMP’s have been identified and screened for phenotypic and mortality effects in *Tribolium castaneum*, of which only two resulted in lethal phenotypes during early and late pupal stages post-injection (46, 47). Thus, targeting of peritrophin genes alone may not be sufficient to achieve high mortality rates, but could be involved in important roles including protection, detoxification, absorption of nutrients, and increasing RNAi efficiency (46, 48, 49). Despite peritrophin silencing making the insect gut more susceptible to chemicals and pathogens affecting their metabolism, growth, and development, these effects might not be as lethal as other genes such as vATPAse, tubulin, or inhibitor of apoptosis (sequence not available). The future publication of a fully annotated *P. japonica* genome will provide better target genes for pest management purposes.

The concentration of body fluid required to degrade 50% of dsRNA (CB50) within one hour is between 45-94 fold lower relative to the CB50’s of *Tribolium castaneum* and *Leptinotarsa decemlineata* (23). These are two model coleopterans that account for a majority of knowledge on how dsRNA impacts beetle species. *P. japonica* has a broad ecological host range, and utilizes a suite of detoxification enzymes induced by feeding to detoxify phytochemicals (35). For these reasons, ingested dsRNA faces a complex biochemical environment in the gut of *P. japonica.* When verifying gene expression, naked dsRNA resulted in no significant difference in expression relative to the non-treated control group. Although ingestion of naked dsRNA did not lead to gene silencing, we observed that the fold change in peritrophin expression in both BAPC-dsRNA treatment groups was significantly lower (*P < 0.05*) compared to the control groups (non-treated control and dsRNA only). These results suggest that increasing molar concentrations of BAPCs (>20 μM) improves efficiency of dsRNA delivery, resulting in the desired biological response. Nonetheless, our previous work with BAPCs in different organisms indicate that BAPCs concentrations >60 μM may trigger cytotoxicity (29, 36). Thus, higher BAPCs concentrations were not tested. While the presence of cationic moieties facilitate binding with the cell membrane, excessive cationic charge can also disrupt cell membrane’s potential and lead to cell death (50–52). Therefore, the optimal dose of BAPCs and any other cationic nanoparticle must be carefully optimized for each nanoparticle to avoid undesirable outcomes. Our results support that BAPC nanoparticles are effective protectants of dsRNA in insect midgut environments and suggest protectants may be required for efficient RNAi in *P. japonica*.

After ingestion, dsRNA passes into the *P. japonica* midgut (15). The midgut is composed of three types of epithelial cells: columnar cells, endocrine cells, and stem cells. Presumably, it is in these cells where dsRNA uptake and processing take place. It has been reported in insects that two main mechanisms are involved in the internalization of dsRNA: receptor mediated uptake or endocytosis. The best documented endocytic route in insects is the clathrin-dependent pathway. In the experiment using 3rd instar larval *P. japonica* midguts in an Ussing chamber, it was observed that fluorescence decreased in the lumen compartment and increased in the hemolymph in a time-dependent manner consistent with our previous reports on transcytosis (29). However, in the presence of CPZ, a clathrin-mediated endocytosis inhibitor, there was a noticeable lack of change in the relative fluorescence in the lumen and smaller increase in hemolymph fluorescence relative to BAPC’s alone. Formation of a clathrin-coated pit is initiated by the rearrangement of various accessory and cytoskeletal proteins along with the creation of a clathrin-coated pit at the inner surface of the cell membrane. CPZ inhibits the anchoring of clathrin and adaptor protein 2 (AP2) complex to endosomes, thereby preventing the assembly of these coated pits (53). This suggests that clathrin-mediated endocytosis may play a significant role in the uptake of NP in the gut, but other pathways are also likely present as some Rh-BAPCs movement was still observed. Despite the increased number of articles demonstrating nanoparticles-dsRNA mediated gene silencing, fundamental mechanisms such as uptake midgut cells or transport to the hemolymph are not widely reported. Thus, our findings are particularly relevant as they suggest mechanisms that could potentially enable systemic delivery or can lead to a more tailored nanoparticle design for gene silencing.

Overall, BAPCs provide a means of reliably protecting dsRNA through oral delivery to *P. japonica*. BAPCs are a new class of biomaterial developed by our research group that stands out in the crowded field of nanoparticle delivery systems due to two crucial features:1) they are assembled exclusively in water, and 2) they contain four free lysine Ɛ-amino groups with pKa values between 9 and 10.5, which makes them stable in neutral and alkaline insect guts. According with DLS, the BAPCs-dsRNA complexes form compact clusters with size ranging from 250 – 350 nm in a pH environment consistent with the gut of *P. japonica* adults. Association of BAPCs with dsRNA confers protection to dsRNA by hidden target sites for RNases, resulting in the stabilization of dsRNA. The use of dsRNA and nanoparticles currently appears expensive when compared with relatively low cost of common insecticides. It is unlikely that dsRNA technology will replace the use of conventional insecticides for the management of *P. japonica.* However, providing targeted control will reduce the negative impacts on non-target arthropods associated with the use of insecticides to control both economically important life stages of *P. japonica*.

## Data availability statement

The raw data supporting the conclusions of this article will be made available by the authors, without undue reservation.

## Author contributions

EC and NK: These authors have contributed equally to this work and share first authorship. Both authors performed survivorship assay, gut dissection for transcytosis experiment and gene expression analysis. In addition, both have equally contributed in preparation of this manuscript. EM: Performed transcytosis experiment on insect midgut tissue. SG: Extracted total RNA from insect gut tissue to synthesize dsRNA for survivorship assay. RR: Provided guidance on primer design, gene expression analysis and data interpretation. JN-N: Helped in synthesis of BAPCs and BAPC-dsRNA formulation as well as their characterization. DH: Provided guidance on planning of insect feeding assay and data collection and interpretation. LA: Supervise the project, provide funds and conceived the original idea of BAPCs use for gene silencing through feeding. All authors contributed to the article and approved the submitted version.
