# Supplementary material for: Gene silencing in adult Popillia japonica through feeding of double‐stranded RNA (dsRNA) complexed with branched amphiphilic peptide capsules (BAPCs)
Source: Front Insect Sci. 2023 May 12;3:1151789. doi: 10.3389/finsc.2023.1151789 (PMC10926504; doi:10.3389/finsc.2023.1151789)
Supplement: Supplementary file 1 [file Image_1.pdf]

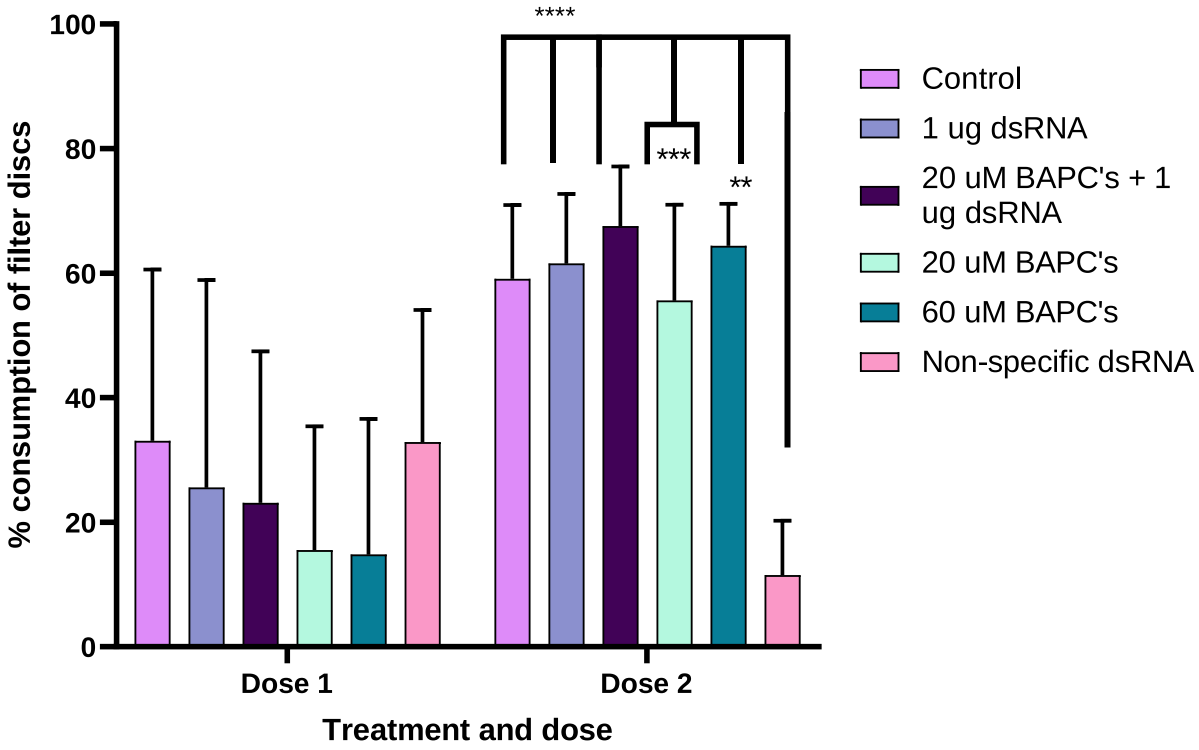

Supplementary Figure 1. Graph illustrating the mean percentage of the filter paper eaten by adult 740 P. japonica within 24 hr. Differences between values were compared by one-way ANOVA using 741 Tukey as a post-test. Statistical significance: (*) P < 0.05; (**) P< 0.01, (***) P< 0.001, (****) 742 P < 0.0001. Non-statistical significance (ns) was considered when P > 0.05.
